# High willingness to use drug consumption rooms among people who inject drugs in Scotland: findings from a national bio-behavioural survey among people who inject drugs

**DOI:** 10.1016/j.drugpo.2020.102731

**Published:** 2021-04

**Authors:** Kirsten M.A. Trayner, Norah E. Palmateer, Sharon J. Hutchinson, David J. Goldberg, Samantha J. Shepherd, Rory N. Gunson, Emily J. Tweed, Saket Priyadarshi, Harry Sumnall, Amanda Atkinson, Andrew McAuley

**Affiliations:** a School of Health and Life Sciences, Glasgow Caledonian University, Glasgow, UK; b Health Protection Scotland, Glasgow, UK; c West of Scotland Specialist Virology Centre, Glasgow, UK; d MRC/CSO Social and Public Health Sciences Unit, University of Glasgow, Glasgow, UK; e NHS Greater Glasgow and Clyde Addictions Services, Glasgow, UK; f Liverpool John Moores University, Liverpool, UK

**Keywords:** Drug consumption rooms, Supervised injection facilities, Harm reduction, People who inject drugs

## Abstract

**Background:**

To address rising drug-related harms (including significant transmission of HIV) among people who inject drugs (PWID) in Glasgow, officials have proposed the introduction of the UK's first drug consumption room (DCR) in Glasgow city centre. Using a nationally representative sample, this study aimed to determine willingness to use a DCR among PWID nationally, in Glasgow city centre (the proposed DCR location), other Scottish city centres (excluding Glasgow) and the rest of Scotland (excluding city centres).

**Methods:**

Bio-behavioural survey, of 1469 current PWID (injected in last 6 months) across Scotland during 2017-18. Willingness to use DCRs was examined by drug-related risk behaviours and harms overall in Scotland, and then stratified by Glasgow city centre (*n =* 219), other Scottish city centres (*n =* 226) and the rest of Scotland (*n =* 1024).

**Results:**

The majority of PWID overall in Scotland (75%) were willing to use a DCR; willingness was higher among those recruited in Glasgow city centre (83%) and other Scottish city centres (83%), compared to the rest of Scotland (72%) (*p* < 0.001). Willingness was greater among PWID who reported (compared to those who did not report) injecting heroin (76%, *p =* 0.002), cocaine injecting (79%, *p =* 0.014), homelessness (86%, *p <* 0.001), public injecting (87%, *p <* 0.001) and an overdose (80%, *p =* 0.026). Willingness was found to be associated with a cumulative multiple risk variable: increased from 66% among those with a score of zero to 85% with a score of at least three (*p <* 0.001).

**Conclusions:**

The vast majority of PWID at greatest risk of drug-related harm in Glasgow and elsewhere in Scotland would be willing to use a DCR, supporting proposals for the introduction of DCRs nationally.

## Introduction

Internationally, there has been an increase in drug-related deaths in recent years, including in Scotland, where figures have reached record levels and are among the highest in Europe (National Records of Scotland, 2019). In addition, there have been recent major outbreaks of infectious disease among people who inject drugs (PWID) in Scotland and the Scottish city of Glasgow; these have included anthrax, the largest outbreak of wound botulism among PWID ever recorded in Europe (Trayner et al., 2018) and the largest outbreak of HIV among PWID observed in the UK for over 30 years, which has resulted in a rapid rise in HIV prevalence from 1% to 11% among PWID in Glasgow city centre (McAuley et al., 2019).

To address the rise in drug-related health burden among PWID in Glasgow, a needs assessment focusing on public drug use was published in 2016, which proposed the establishment of the UK's first drug consumption room (DCR) and co-located heroin assisted treatment (HAT) service located in Glasgow city centre (Tweed, Rodgers, Priyadarshi, & Crighton, 2018). The HAT service was established in November 2019, embedded in a service providing care for those experiencing homelessness. However, despite attracting widespread support from Scottish Government, local authorities, health officials, some sections of the media (Atkinson, McAuley, Trayner, & Sumnall, 2019), and police, the proposals for a DCR have been repeatedly rejected by the UK Government, on the basis of a lack of an appropriate legal framework for the facility to operate under the Misuse of Drugs Act 1971. The UK Government reiterated its objection to DCRs at the UK Drug-Related Death's summit, held in Glasgow on the 27^th^ of February 2020 (BBC news, 2020).

Drug consumption rooms are healthcare settings, which provide a safe and clean environment for the consumption of drugs under the supervision of medically trained staff and alongside provision of clean injecting equipment. There are over 100 DCRs operating internationally, which are typically low threshold interventions that aim to attract PWID at the highest risk of drug-related and social harm (Pardo, Caulkins, & Kilmer, 2018). There is a large body of literature which supports the introduction of DCRs, with a particularly strong case for areas of the world, such as Glasgow, which are undergoing significant public health crises among PWID. Published studies suggest that DCRs are successful in attracting the most marginalised PWID, reduce overdose morbidity and mortality, promote safer injection conditions and enhance access to health and social services (Caulkins, Pardo, & Kilmer, 2019; Pardo et al., 2018).

Reluctance to implement DCRs in Scotland may be, in part, hindered by lack of evidence on the willingness of PWID to use the service. Internationally, a number of studies have assessed the acceptability of DCRs among PWID at a local or regional level (Bouvier, Elston, Hadland, Green, & Marshall, 2017; Butler, Chapman, & Terry, 2018; Fry, Fox, & Rumbold, 1999; Green, Hankins, Palmer, Boivin, & Platt, 2004; O'Rourke et al., 2019). Willingness to use DCRs has been associated with drug-related risk factors such as public injecting, homelessness, cocaine injecting, history of overdose and frequent drug use (Bouvier et al., 2017; Green et al., 2004; O'Rourke et al., 2019). We here assess willingness to use a DCR among a large national sample of PWID, overall in Scotland and also stratified by Glasgow city centre (location of an ongoing outbreak of HIV and planned location of the DCR), Scottish city centres (excluding Glasgow) and the rest of Scotland. This study therefore aimed to investigate willingness to use a DCR among PWID Scotland, in light of the proposals to establish the UK's first DCR.

## Methods

### Data source

This research utilised a national anonymous, bio-behavioural, cross-sectional survey known as the Needle Exchange Surveillance Initiative (NESI). NESI aims to measure the prevalence of blood-borne viruses (BBV) and injecting risk behaviours among PWID across mainland Scotland. Trained independent interviewers recruited PWID from over 100 services providing injecting equipment and other harm reduction interventions (approximately half of the total number of services across Scotland). Inclusion of PWID who have not injected recently (in past six months) is capped at 30% to ensure that the majority of the sample represents recent PWID (defined as those who injected in the past six months). After providing informed consent, participants complete an interviewer-led questionnaire and then provide a dried blood spot (DBS) sample, which is tested anonymously for BBV markers as described elsewhere (Health Protection Scotland, 2019). A question relating to willingness to use a DCR was included in the survey conducted between July 2017 and October 2018. Thus, this analysis was confined to recent PWID (injected in last six months) surveyed during 2017-18.

### Measures

The primary outcome of interest was willingness to use a DCR. This was ascertained by providing a short description of a DCR: “A drug consumption room is a place where you can bring your drugs to inject or smoke in a safe environment. You can also get clean works, help with injecting technique and other advice”, followed by the question “would you use a drug consumption room if it were made available in your area?” (answered yes/no). Willingness was explored according to demographic, injecting risk behaviour and harm variables. Exposures of interest (in the last six months unless stated otherwise) included: age, sex, homelessness (yes/no), injected heroin (yes/no), injected powder cocaine (yes/no), injected in a public place (yes/no), HIV infection, current HCV infection, overdosed in the last year (yes/no) and skin and soft tissue infection in the last year (SSTI) (yes/no). HIV and HCV infection were confirmed by laboratory DBS tests. Samples which tested both HCV antibody and PCR positive represented those with current HCV infection. A multiple risk variable was calculated as the sum of the following individual risk factors: cocaine injecting, injecting in a public place, sharing needles/syringes, high injecting frequency (≥4 times per day), and re-using needles/syringes. A participant received a score of one for each risk factor that was present, with a maximum score of five (those with a score of 3, 4 or 5 were grouped given too few observations).

### Analysis

Proportions of those willing to use a DCR were compared across demographic, injecting risk behaviour and harm variables using chi-squared tests of association across the total Scottish sample. Given proposals to establish a DCR in Glasgow city centre, we further stratified the analysis by; Glasgow city centre (*n =* 219), other Scottish city centres (excluding Glasgow) (hereafter “Scottish city centres”) (*n =* 226) and rest of Scotland (excluding city centres) (hereafter “rest of Scotland”) (*n =* 1024). We used a significance level of *p <* 0.05.

Any co-variates which were associated with willingness to use a DCR (in overall or stratified analysis) at a univariate level were included in a logistic regression model. We included: recruitment region, homelessness, injecting heroin, reporting an overdose in the last year and our multiple risk variable. Variables which were already included in the multiple risk variable (cocaine injecting and public injecting) were not included in the model. The results of the model are described in the results; the full model can be viewed in the Appendix. Analysis was undertaken using Stata 13.

## Results

Our sample included 1469 PWID, 15% (219/1469) were recruited in Glasgow city centre, 15% (226/1469) in Scottish city centres and 70% (1024/1469) in the rest of Scotland. The majority were male (75%, 1099/1465) and aged 35-44 years old (52%, 769/1468). Just over a quarter had experienced recent homelessness (27%, 402/1467). The vast majority (92%, 1347/1465) had injected heroin in the last six months and 31% (452/1464) had injected powder cocaine. The prevalence of HIV and current HCV among participants was 3% (42/1367) and 33% (403/1247), respectively. In the last year, 18% (265/1437) had experienced an overdose and 28% (402/1456) an SSTI. A higher proportion of PWID recruited in Glasgow city centre reported recent homelessness (57%, 124/218), cocaine injecting (74%, 161/219) and public injecting (47%, 102/219) when compared to other regions (Table 1).

**Table 1:** Willingness to use a Drug Consumption Room (DCR) among 1469 PWID Scotland during 2017-18, stratified by Glasgow City Centre (proposed location of the first DCR), Scottish city centres and rest of Scotland, according to demographic factors, injecting risk behaviours, and drug-related harms, 2017–18.

	All Scotland			Glasgow city centre			Scotland city centres (excluding Glasgow)			Rest of Scotland (excluding city centres)		
	Willing to use a DCR^a^, (n/N)	95% CI	*P*-value^b^^,^^c^	Willing to use a DCR^a^, (n/N)	95% CI	*P*-value^b^^,^^c^	Willing to use a DCR^a^, (n/N)	95% CI	*P*-value^b^^,^^c^	Willing to use a DCR^a^, (n/N)	95% CI	*P*-value^b^^,^^c^
***Demographics***												

**Total (27 non-responses regarding willingness to use a DCR) (%)**	75% (1082/1442)	73–77		83% (179/216)	77–87	N/A	83% (184/223)	77–87	N/A	72% (719/1003)	69–74	N/A
**Gender (4 non-responses)**^d^												
Male	76% (817/1080)	73–78		84% (154/184)	77–88		81% (141/174)	75–86		72% (522/722)	69–75	
Female	73% (262/358)	68–78	0.351	77% (24/31)	60–89	0.392	88% (43/49)	75–94	0.274	70% (195/278)	65–75	0.498
**Age (1 non-response)**												
<35	77% (279/364)	72–81		83% (30/36)	68–92		85% (70/82)	75–94		73% (179/246)	67–78	
35–44	75% (563/752)	72–77		86% (100/116)	78–91	0.258	82% (90/110)	74–88		71% (373/526)	67–75	
44+	74% (239/325)	69–78	0.636	77% (49/64)	64–85		77% (24/31)	60–89	0.590	72% (166/230)	66–78	0.851
**Homeless in last 6 months (2 non-responses)**												
Yes	86% (340/394)	83–89		87% (108/124)	80–91		92% (72/78)	84–96		83% (160/192)	77–88	
No	71% (740/1046)	68–73	**<0.001**	77% (70/91)	67–84	**0.051**	77% (112/145)	70–83	**0.005**	69% (558/810)	66–72	**<0.001**

***Injecting risk behaviours***												

**Injected heroin in the last 6 months (4 non-responses)**												
Yes	76% (1005/1322)	74–78		85% (157/184)	80–90		85% (178/210)	79–89		72% (670/928)	69–75	
No	63% (74/117)	54–71	**0.002**	69% (22/32)	51–82	**0.022**	36% (4/11)	15–64	**<0.001**	65% (48/74)	53–75	0.178
**Injected cocaine in the last 6 months (5 non-responses)**												
Yes	79% (353/446)	75–83		84% (133/158)	78–89		75% (24/32)	58–87		77% (196/256)	71–81	
No	73% (725/992)	70–76	**0.014**	79% (46/58)	67–88	0.400	84% (158/189)	78–88	0.238	70% (521/745)	67–73	**0.042**
**Injected in a public place in last 6 months (5 non-responses)**												
Yes	87% (203/233)	82–91		86% (87/101)	78–92		95% (36/38)	83–99		85% (80/94)	77–91	
No	72% (877/1206)	70–75	**<0.001**	80% (92/115)	72–86	0.232	80% (146/183)	73–85	**0.028**	70% (639/908)	67–73	**0.003**
**Multiple risk variable**^e^**^,^**^f^**(28 non-responses)**												
0	66% (267/404)	61–70		56% (10/18)	34–75		71% (45/63)	59–81		66% (212/323)	60–71	
1	75% (377/501)	71–79		88% (43/49)	76–94		83% (72/87)	74–89		72% (262/365)	67–76	
2	80% (273/341)	76–84		81% (57/70)	71–89		90% (45/50)	79–96		77% (171/221)	71–82	
3, 4 or 5	85% (147/172)	79–90	**<0.001**	86% (63/73)	77–92	**0.013**	95% (19/20)	76–99	**0.025**	82% (65/79)	72–89	**0.003**

***Harms***												

**HIV infection**												
Yes	85% (35/41)	72–93		92% (24/26)	76–98		*	10–91		77% (10/13)	50–92	
No	74% (967/1300)	72–77	0.111	81% (153/188)	75–86	0.167	*	77–88	0.218	71% (652/917)	68–74	0.645
**Current HCV infection**												
Yes	77% (307/399)	73–81		85% (79/93)	76–91		88% (45/51)	77–95		72% (183/255)	66–77	
No	74% (613/825)	72–77	0.316	83% (87/105)	75–89	0.690	79% (98/124)	71–85	0.152	72% (428/596)	86–91	0.989
**Overdosed in the last year (32 non-responses)**												
Yes	80% (210/261)	75–85		82% (46/56)	70–90		90% (46/51)	79–96		77% (118/154)	69–83	
No	74% (857/1160)	71–76	**0.026**	83% (131/157)	77–88	0.824	80% (133/166)	73–86	0.098	71% (593/837)	68–74	0.144
**Skin and soft tissue infection in the last year (13 non-responses)**												
Yes	78% (310/397)	74–82		81% (63/78)	71–88		83% (64/77)	73–90		76% (183/242)	70–81	
No	74% (772/1044)	71–77	0.105	84% (116/138)	77–89	0.538	81% (120/146)	75–88	0.863	71% (536/760)	67–74	0.125

a May not add up due to missing data;

b Derived from chi-squared test;

c Missing values excluded from analysis;

d Non-responses/missing data is only presented if it is greater than 5% of the total sample;

e Comprised of the following risk factors: cocaine injecting, public injecting, sharing needles/syringes, high injecting frequency and re-using needle/syringes,

f Chi-squared test for trend,

⁎ Data suppressed to prevent deductive disclosure.

We found overall willingness to use a DCR in Scotland was 75%; willingness was higher among those recruited in Glasgow city centre (83%) and other Scottish city centres (83%), compared to the rest of Scotland (72%) (*p <* 0.001). We found a higher willingness among PWID who reported injecting heroin (76%) compared to those who did not (63%) (*p =* 0.002), with a similar result across all stratified regions. Similarly, those who reported recent homelessness in Scotland (86%), reported a higher willingness compared to those who did not (71%, *p <* 0.001), which was found across all regions. Other risk factors where PWID reported a higher willingness were those who reported public injecting (87% vs. 72%, *p <* 0.001), cocaine injecting (79% vs. 73%, *p =* 0.014) and reporting an overdose in the last year (80% vs. 74%, *p =* 0.026), with similar rates found across all regions (Table 1).

An increasing significant positive trend was found across all regions in relation to an increasing score in our cumulative risk variable. Among the total sample, PWID with the lowest score (0) had the lowest willingness (66%), compared to 85% (*p <* 0.001) with the highest (score of three, four or five) (Table 1).

In our multi-variate analysis, PWID recruited in Scottish city centres (aOR=1.62, 95% CI 1.10 to 2.39, *p =* 0.014) had a significantly higher odds of reporting willingness when compared to individuals recruited from the rest of Scotland. Those who reported homelessness (relative to those who did not) (aOR=2.06, 95% CI 1.47 to 2.89, *p <* 0.001) and injecting heroin (compared to those who did not) (aOR=2.07, 95% CI 1.35 to 3.18, *p =* 0.001) were associated with an increased odds of reporting willingness to use a DCR. When compared to a baseline score of 0, all scores in our cumulative risk variable were associated with increased odds of reporting willingness. The highest odds were found (aOR=2.29, 95% CI 1.37 to 3.83, *p =* 0.001) among PWID who had a score of 3, 4 or 5, when compared to those who had a score of 0 (Appendix 1).

## Discussion

In the context of proposals to establish the UK's first DCR in Glasgow, we found that the vast majority (75%) of PWID in Scotland expressed willingness to use a DCR. The high level of willingness to use a DCR among PWID in Scotland is consistent with other studies which have been conducted in cities elsewhere in the UK (London and Leeds) (84-89%) (Butler et al., 2018; Hunt, Lloyd, Kimber, & Tompkins, 2007) and in North America (76-87%) (Bouvier et al., 2017; Green et al., 2004). Furthermore, studies in cities which have successfully introduced DCRs, such as Vancouver (92%) (Kerr, Wood, Small, Palepu, & Tyndall, 2003) and Melbourne (77%) (Fry et al., 1999) showed similar acceptability rates prior to their introduction.

Previous research has shown that the voices of PWID have been left out of the debate for a DCR in Glasgow (Atkinson et al., 2019), until now. We found a particularly high willingness among those recruited in Glasgow city centre (83%), which may reflect increased awareness of DCRs in Glasgow, given the local proposals, where individuals may have had more time to consider the benefits/drawbacks. However, PWID recruited in other Scottish city centres (83%) also reported high willingness which is an important result, as DCRs are normally established in cities, close to open drug markets that are experiencing issues with public drug use. These results are also encouraging given the planned location of the DCR in Glasgow (Tweed et al., 2018), and for any other cities in Scotland which may consider introducing DCRs in the future.

Outside of city centres, willingness to use a DCR in the rest of Scotland was also high (72%), one reason for this may be related to contemporaneous media interest and reporting on DCRs in the UK (Atkinson et al., 2019). If a change in legal framework allowed for the introduction of DCRs, services (such as mobile DCRs) would need to be considered to meet the demand outside of city centres. Assessing self-reported willingness to use DCRs has been shown to be a good predictor of subsequent attendance and use of DCRs; a study from Vancouver reported that 72% of PWID who reported prior willingness later used the service (DeBeck et al., 2012).

Key individual and environmental drivers of an increase in HIV prevalence among PWID in Glasgow in recent years are a combination of homelessness, cocaine injecting and public injecting (McAuley et al., 2019; Trayner et al., 2020). We found a high willingness among PWID reporting these risk factors (at levels of 79%-87% nationally, 84-87% in Glasgow city centre, 75-92% in other Scottish city centres and 77-85% in the rest of Scotland). Moreover, we found a positive association between cumulative risk and willingness, with willingness to use a DCR increasing with a higher score in our cumulative risk variable (based on self report of public injecting, cocaine injecting, sharing injecting equipment, re-using injecting equipment and a high injecting frequency). Our results indicate that if a DCR were piloted in Glasgow as proposed, or elsewhere in Scotland, it would likely attract PWID with the greatest risk of drug-related harm, consistent with previous research (Bouvier et al., 2017; Butler et al., 2018; Fry et al., 1999; Green et al., 2004; Wood et al., 2003).

Cities such as Vancouver were faced with similar health crises prior to the establishment of DCRs. For example, before the establishment of Insite (North Americas first DCR), Vancouver experienced a major outbreak of HIV in the mid-1990s, and this was also driven by cocaine injecting and homelessness (Tyndall et al., 2003). In addition to high levels of public injecting, these were the main drivers for the establishment of the service. Evaluations of InSite showed that the number of PWID injecting in public halved within 12 weeks of the facility opening (Wood et al., 2004) and a mathematical modelling study predicted that 35 new HIV cases were prevented each year (Andresen & Boyd, 2010). Furthermore, the fatal overdose rate decreased by 35% within 500m of the DCR (Marshall, Milloy, Wood, Montaner, & Kerr, 2011). Given the high willingness to use DCRs among high risk PWID in our survey, our results suggest that the introduction of a DCR in Glasgow or elsewhere could potentially yield similar results.

With regard to limitations, all data collected through NESI (apart from BBV) status) is self-reported, which may be subject to response and recall bias. However, the likelihood of response bias is minimised to a certain extent by the use of independent researchers to collect data. Additionally, a limitation is that these results are in the context of the population having no experience of DCRs and thus may not have been aware of DCRs prior to their interview. Our data relate to those who attend services that provide injecting equipment, and thus may not fully represent the PWID population in Scotland; however, other data from elsewhere in Scotland (Tweed et al., 2018) highlighted that the majority of PWID are regular attendees of these services. Furthermore, data was collected from 139 services providing injecting equipment (approximately half of the total nationally) and our sample represents an estimated 10-15% of the PWID population in Scotland.

## Conclusion

The case for DCRs in Scotland, based on the extent of drug-related harms alone, is compelling. We now provide further evidence to demonstrate that the vast majority of PWID, particularly those most at risk of drug-related harm, in Glasgow and indeed elsewhere in Scotland would be willing to use a DCR.

## Declarations of Interest

SJH has received honoraria from Gilead, unrelated to this study. SP is the project lead on the proposal for the described drug consumption room in Glasgow city centre. All remaining authors have nothing to disclose.

## Appendix Supplementary materials

Image, application 1
