# Supplementary material for: High willingness to use drug consumption rooms among people who inject drugs in Scotland: findings from a national bio-behavioural survey among people who inject drugs
Source: Int J Drug Policy. 2021 Apr;90:102731. doi: 10.1016/j.drugpo.2020.102731 (PMC8063177; doi:10.1016/j.drugpo.2020.102731)
Supplement: Supplementary file 1 [file mmc1.docx]

| **Appendix. Odds ratios (OR), adjusted odds ratios (aOR) and 95% confidence intervals (CI) of factors associated with willingness to use a Drug Consumption Room (DCR) among current PWID (injected in last six months) in Scotland, 2017-18** | | | | | | |
| --- | --- | --- | --- | --- | --- | --- |
|  |  |  |  |  |  |  |
|  | **Total^a,^ N** | **Willing to use a DCR^b^, (% of N)** | **Overall sample (n=1442, 1082 willing to use a DCR)** | | | |
|  |  |  | **OR (95% CI)** | **p-value** | **aOR (95% CI)** | **p-value** |
| **Region recruited** |  |  |  |  |  |  |
| Rest of Scotland (excluding city centres) | 1003 | 719 (72) | 1 |  | 1 |  |
| Scottish city centres (excluding Glasgow) | 223 | 184 (83) | 1.86 (1.28 to 2.70) | 0.001 | 1.62 (1.10 to 2.39) | 0.014 |
| Glasgow city centre | 216 | 179 (83) | 1.91 (1.31 to 2.79) | 0.001 | 1.19 (0.78 to 1.83) | 0.409 |
| **Homeless in last 6 months** |  |  |  |  |  |  |
| No | 1046 | 740 (71) | 1 |  | 1 |  |
| Yes | 394 | 340 (86) | 2.60 (1.89 to 3.57) | <0.001 | 2.06 (1.47 to 2.89) | <0.001 |
| **Injected heroin in last 6 months** | |  |  |  |  |  |
| No | 117 | 74 (63) | 1 |  | 1 |  |
| Yes | 1322 | 1005 (76) | 1.84 (1.23 to 2.74) | 0.003 | 2.07 (1.35 to 3.18) | 0.001 |
| **Overdosed in the last year** | |  |  |  |  |  |
| No | 1160 | 857 (74) | 1 |  | 1 |  |
| Yes | 261 | 210 (80) | 1.46 (1.04 to 2.03) | 0.027 | 1.04 (0.72 to 1.48) | 0.832 |
| **Multiple risk variable^c^** |  |  |  |  |  |  |
| 0 | 404 | 267 (66) | 1 |  | 1 |  |
| 1 | 501 | 377 (75) | 1.56 (1.17 to 2.08) | 0.003 | 1.45 (1.08 to 1.95) | 0.015 |
| 2 | 341 | 273 (80) | 2.05 (1.47 to 2.88) | <0.001 | 1.96 (1.37 to 2.81) | <0.001 |
| 3, 4 or 5 | 172 | 147 (85) | 3.02 (1.88 to 4.83) | <0.001 | 2.29 (1.37 to 3.83) | 0.001 |
|  |  |  |  |  |  |  |
| ^a^ Excludes missing data |  |  |  |  |  |  |
| ^b^ May not add due to missing data |  |  |  |  |  |  |
| ^c^ Comprised of the following risk factors: cocaine injecting, public injecting, sharing needles/syringes, high injecting frequency and re-using needle/syringes | | | | | |  |
